# Antibacterial Enhancement of High-Efficiency Particulate Air Filters Modified with Graphene-Silver Hybrid Material

**DOI:** 10.3390/microorganisms11030745

**Published:** 2023-03-14

**Authors:** Alexandra Ciorîță, Maria Suciu, Maria Coroş, Codruța Varodi, Florina Pogăcean, Lidia Măgeruşan, Valentin Mirel, Raluca-Ioana Ștefan-van Staden, Stela Pruneanu

**Affiliations:** 1 Faculty of Biology and Geology, Babeș-Bolyai University, 44 Republicii Street, 400015 Cluj-Napoca, Romania; 2 National Institute for Research and Development of Isotopic and Molecular Technologies, 67-103, Donat Street, 400293 Cluj-Napoca, Romania; 3 Laboratory of Electrochemistry and PATLAB, National Institute of Research for Electrochemistry and Condensed Matter, 202 Splaiul Independentei Street, 060021 Bucharest, Romania

**Keywords:** graphene, silver nanoparticles, Gr-Ag (5 wt% Ag) hybrid, modified HEPA filter, antibacterial effect

## Abstract

Bacterial infections are a major concern as antibiotic resistance poses a great threat, therefore leading to a race against time into finding new drugs or improving the existing resources. Nanomaterials with high surface area and bactericidal properties are the most promising ones that help combating microbial infections. In our case, graphene decorated with silver nanoparticles Gr-Ag (5 wt% Ag) exhibited inhibitory capacity against *S. aureus* and *E. coli*. The newly formed hybrid material was next incubated with high-efficiency particulate air (HEPA) filter, to obtain one with bactericidal properties. The modified filter had greater inhibitory action against the tested strains, compared to the control, and the effect was better against the Gram-negative model. Even if the bacteria remained attached to the filters, their colony forming unit capacity was affected by the Gr-Ag (5 wt% Ag) hybrid material, when they were subsequently re-cultured on fresh agar media. Therefore, the HEPA filter modified with Gr-Ag (5 wt% Ag) has high antibacterial properties that may substantially improve the existing technology.

## 1. Introduction

Before the antibiotic era began, bacterial infections were of major concern worldwide. A few decades after the discovery of antibiotics, a new problem arises: antibiotic-resistant bacteria. Issues such as uncontrolled administration of antibiotics most probably created this inconvenience, but a few years back, a study showed that bacterial resistance to antibiotics is an ancient adaptation, that appeared long before the discovery of antibiotics [1]. With this information at hand, the struggle continues in fighting bacterial diseases with new or improved technologies [2,3,4].

A well-known application is nanotechnology and its implication in the medical field, due to the development of 2D and 3D nanomaterials with wide versatility [5]. Such nanomaterials are nanoparticles (NPs), within the range of 1 and up to 100 nm [5,6,7], and graphene nanosheets (linear size from tens of nm to µm). In recent years, the interest in graphene-based materials reached many complementary domains such as chemistry [8,9], biomedicine [10,11] and biotechnology [12], but also in material sciences [13], automotive [14], alternative energies [15] and other industries [16,17,18]. These 2D nanomaterials are a promising solution for combating bacterial infections and preventing biofilm formation, because of the large surface area that they cover, easy surface functionalization and low necessary doses of administration [19].

In addition, the importance and environmental impact of silver has been intensely studied in the last decades [20] and although it is futile, but worth mentioning its antibacterial properties are thoroughly documented [5,21,22,23,24]. Therefore, combining the properties of graphene-based nanomaterials and silver nanoparticles could enhance the antibacterial effect and improve the action (lower doses, lower exposure periods, etc.) [8,25].

The aim of this study was to develop a graphene-silver (Gr-Ag) hybrid material, with enhanced antibacterial properties and multi-factorial applications. To achieve the former, high-efficiency particulate air (HEPA) filters were used. HEPA filters are generally employed as a particulate containment device in ventilation systems and in nuclear facilities, capable of removing 0.3 µm particles, with high efficiency (>99%) [26,27]. Therefore, treating HEPA filters with the Gr-Ag hybrid material obtained herein, an existing technology was improved with the use of a modern and eco-friendly approach of nanotechnology.

## 2. Materials and Methods

### 2.1. Chemicals

All reagents used for Gr-Ag (3, 5 and 20 wt% Ag) hybrids synthesis were of analytical grade and used without further purification. Silver nitrate and sodium ascorbate were purchased from Merck (Darmstadt, Germany). The reagents used for the antibacterial assays and SEM preparation were acquired from Sigma-Aldrich Chemie GmbH (Steinheim, Germany). The Mueller-Hinton broth was acquired from Oxoid (Thermo Fisher Scientific Inc., Kandel, Germany), the agar powder was acquired from VWR Chemicals (VWR International, Darmstadt, Germany) and ciprofloxacin of 98% purity was acquired from Alfa-Aesar (Thermo Fisher Scientific Inc., Kandel, Germany). The filter discs used in the agar-well diffusion method were Whatman AA grade (6 mm discs). The HEPA filter (DREISSNER-K0089DREIS) was bought from Germany. Colloidal silver nanoparticles (AgNPs) with the mean size of 9 nm were synthesized by Pure Life SRL (Suceava, Romania) and bought from a local drugstore.

### 2.2. Apparatus

The morphological investigation of AgNPs, reduced graphene oxide (rGO), Gr-Ag (3, 5 and 20 wt% Ag) hybrids, and HEPA filter modified with Gr-Ag (5 wt% Ag) was conducted using the TEM/STEM Hitachi HD-2700 cold-field emission, operated at 200 kV (Hitachi, Tokyo, Japan). The structural characteristics of Gr-Ag (5 wt% Ag) hybrid material were studied by X-ray powder diffraction (XRD). The pattern was recorded with DIFFRAC plus XRD Commander Package on a Bruker-D8 Advance Diffractometer with the tube set at 40 kV and 40 mA. A germanium (1 1 1) monochromator was placed in the incident beam (λ = 1.54056 Å) and the scan rate was 0.02 s^−1^. Raman spectroscopy was performed on Gr-Ag (5 wt% Ag) hybrid material using an NTEGRA Spectra platform, placed on a NEWPORT RS4000 optical table with a vibration isolation system and equipped with a SOLAR TII confocal Raman spectrometer coupled with an Olympus IX71 microscope in two different configurations. Detection was achieved with a CCD camera (NT-MDT; Moscow, Russia). FTIR measurements (4000–400 cm^−1^) were recorded with a Bruker Tensor II spectrometer (Esslingen, Germany), with the Gr-Ag (5 wt% Ag) hybrid material embedded in KBr pellet.

### 2.3. Synthesis of Reduced Graphene Oxide (rGO)

Graphene oxide (GO) was thermally reduced to prepare the reduced graphene oxide (rGO) flakes, as previously described [28]. Briefly, 200 mg GO were placed into a quartz boat and put in a temperature-programmed oven. GO was reduced by keeping the temperature at 250 °C for 5 min, under argon flow (0.1 L/min). The heating rate was 10 °C/min. Representative TEM micrographs of rGO can be seen in Figure S1—Supporting Material. The micrographs reveal that the graphene flakes are very thin and exhibit a wrinkled surface.

### 2.4. Synthesis of Gr-Ag (3, 5 and 20 wt% Ag) Hybrid Materials

GO was prepared from graphite powder (flake size < 20 μm, from Sigma Aldrich) using a modified Hummers method, as previously described [29]. Three Gr-Ag hybrid materials were synthesized and the Ag content was selected to be 3, 5 and 20 wt%. The synthesis was performed as following described. A quantity of 700 mg of GO powder was dispersed in 180 mL H_2_O and sonicated for 30 min to obtain a homogeneous GO dispersion. Next, AgNO_3_ (33 mg for 3 wt% Ag, 55 mg for 5 wt% Ag and 220 mg for 20 wt% Ag) previously dissolved in H_2_O (~100 mL) was added to the mixture and stirred at room temperature, until the next day. After that, a solution of sodium ascorbate (1800 mg in 20 mL H_2_O) was added in order to simultaneously reduce the silver ions and graphene oxide. The mixture was following heated at 100 °C and maintained at constant temperature for 3 h. The resulting homogeneous black suspension was then filtered, washed with water several times, and finally dried by lyophilization. Representative TEM images of Gr-Ag (3, 5 and 20 wt% Ag) hybrid materials are shown in Figure S2a,c,e, respectively. In all cases, one can see the presence of graphene flakes with irregular shapes and the attached silver nanoparticles (dark spots). As expected, the graphene surface is not smooth, presenting many wrinkles. The size distribution of AgNPs is also shown here, being: 24.85 ± 8.3 nm (3 wt% Ag); 47.26 ± 17.93 nm (5 wt% Ag) and 27.39 ± 11.43 nm (20 wt% Ag). In all cases, the number of counted particles was 300.

### 2.5. Antibacterial Characterization of rGO, AgNPs, and Gr-Ag Hybrid Materials

The antibacterial activity of rGO, AgNPs and Gr-Ag (3, 5 and 20 wt% Ag) hybrid materials were assessed against two bacterial strains (*S. aureus* ATCC 25923 and *E. coli* ATCC 25922), through the agar-well diffusion method. Petri dishes with Mueller-Hinton (MH) agar media were inoculated for 24 h with bacterial strains at 0.5 McFarland turbidity standards, as indicated by the EUCAST protocols [30]. The plates were incubated for 1 h at 37 °C to allow the bacterial suspension to infiltrate the media. After this, 6 mm wells were carved in the agar media using a sterile cork. The wells were filled with Whatman filter discs to prevent the solutions/powders from dispersing underneath the media. One plate contained a positive control (10 µg/mL ciprofloxacin), a vehicle control (ethanol) and three wells with Gr-Ag powder (50 µL of 10 mg/mL concentration), one plate contained three wells with rGO (50 µL of 10 mg/mL), and one plate contained three wells with AgNPs (50 µL of ~11 mg/mL). The plates were incubated for 24 h, at 37 °C after which the inhibition zone formed, indicating the antibacterial action, was measured. The experiment was conducted in duplicate (unless otherwise stated) and the mean value was calculated.

### 2.6. Preparation of HEPA Filter Modified with Gr-Ag (5 wt% Ag) Hybrid Material

Three solutions containing various concentrations (1, 2 and 3 mg/mL) of Gr-Ag (5 wt% Ag) hybrid material were prepared in pure ethanol (6 mL final volume for each concentration). After preparation, the solutions were homogenized by ultrasound, for 10 min. In each solution, one piece of HEPA filter (2 × 2 cm^2^) was immersed for 1 h. After that, the pieces were removed from the solutions and dried at room temperature until the next day. As control, one piece of HEPA filter (without Gr-Ag) was employed. It was also immersed in pure ethanol (1 h) then dried at room temperature for 24 h. Based on the observed results concerning the antibacterial activity, a new series of HEPA filters were prepared, where the immersion time was varied (15, 30, 45 and 60 min). The filters thus prepared were examined at SEM Hitachi SU8230 (Hitachi, Tokyo, Japan).

### 2.7. Microbiological Investigation of HEPA Filter Modified with Gr-Ag (5 wt% Ag) Hybrid Material

The antibacterial activity of the HEPA filter modified with Gr-Ag (5 wt% Ag) hybrid material was assessed according to the EUCAST protocols. Hence, the filters were adjusted to the same size (~1 cm^2^), placed in 6 or 12 well plates, and left for 3 h in PBS (phosphate buffer saline, 0.1 M) under UV light to increase their hydrophobicity. After this period, 1 mL of each bacterial suspension (0.5 McFarland turbidity) was inoculated in each well containing the HEPA filters treated with different concentrations of Gr-Ag (5 wt% Ag) hybrid material for different time periods. Each plate contained a negative control (bacterial suspension without HEPA filters), and were incubated for 24 h at 37 °C. From each well, 100 µL of suspension were taken and diluted up to 10^10^ in PBS. Next, 1 mL of bacterial suspension was inoculated on new plates with MH agar media for colony forming units (CFU) determination. Additionally, to determine if the bacteria remained on the filters, three different protocols were used. Samples were cut from the filters and placed on Petri dishes with MH agar media, incubated at 37 °C, and interpreted after 24 h. From the remaining filters, small pieces were prepared for SEM analysis as following: 2 h treatment with 3% GTA (glutaraldehyde), four washes with PBS (1 h each), dehydration with acetone in increasing concentrations (30%, 50%, 100%), and post fixation with hexamethyldisilazane (1:2, 1:1 and 1:0 with acetone) for 3 h. The samples were analyzed using the SEM Hitachi SU8230, operated at 30 kV.

### 2.8. Statistical Analyses

Students’ *t* test was performed to assess the level of significance of the antibacterial effects observed against *E. coli* and *S. aureus*. The analysis was performed with the help of Origin 8 Software (Origin Lab Corporation, Northampton, MA, USA).

## 3. Results

### 3.1. Antibacterial Activity of the Tested Materials (rGO, AgNPs, Gr-Ag)

The inhibitory capacity of bacterial growth of rGO, AgNPs and three combinations of both materials (3, 5 and 20 wt% Ag) were assayed. The results showed that rGO has the capacity to inhibit only *E. coli*, while AgNPs have inhibitory effects against both bacterial strains. As can be seen in Figure S3, the Gr-Ag (3 wt% Ag) hybrid material had inhibitory effects against *S. aureus* only, while the Gr-Ag (5 wt% Ag) and Gr-Ag (20 wt% Ag) were efficient against both *E. coli* and *S. aureus*. Since the first hybrid material at which the inhibition of both bacterial strains (inhibition zone diameter greater than 7 mm) occurred was Gr-Ag (5 wt% Ag), this one was considered optimal for further tests. Thus, The Gr-Ag (5 wt% Ag) hybrid material was next used for modification of the HEPA filter, in order to increase its antibacterial properties.

There are a few advantages in using the Gr-Ag (5 wt% Ag) hybrid rather than AgNPs. AgNPs have better antibacterial properties in comparison with graphene but by combining the two materials the effect is amplified (Figure S3). In addition, the amount of AgNPs employed for such purposes can be reduced, which is very advantageous from the economic point of view.

### 3.2. Structural Characterization of Gr-Ag (5 wt% Ag) Hybrid Material 

**Figure 1:**
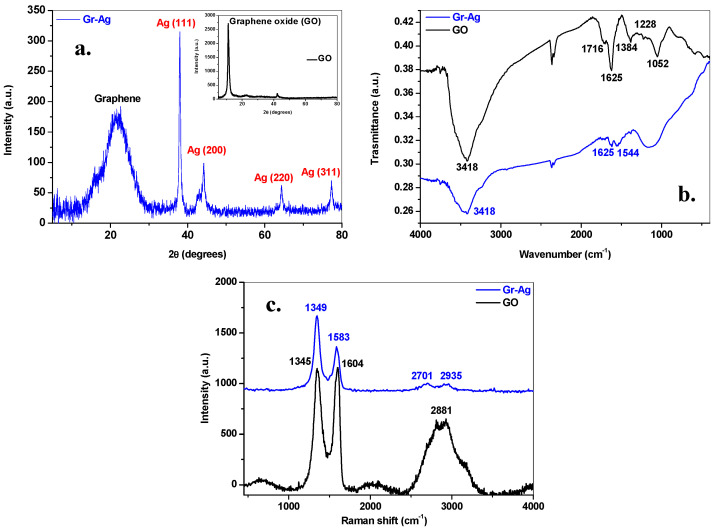
The XRD pattern of Gr-Ag (5 wt% Ag) hybrid material revealing the characteristic peaks of graphene and silver (**a**); Inset: the XRD pattern of graphene oxide (GO); The FTIR spectrum of GO (black line) and Gr-Ag (5 wt% Ag; blue line) (**b**); Raman spectrum of GO (black line) and Gr-Ag (5 wt% Ag; blue line) (**c**).

To structurally characterize the Gr-Ag (5 wt% Ag) hybrid material having the optimal antibacterial properties, various techniques were employed such as XRD, FTIR and Raman. Figure 1a presents the XRD pattern of the synthesized material which exhibits typical diffraction lines both for the graphene and silver. The broad peak centered at approximately 22° is assigned to (002) reflections of graphene. From the XRD pattern three types of structural information were obtained: the number of graphene layers (n), the interlayer distance (d) and the size of graphene crystallites (D). In this case, the number of layers was 3, the interlayer distance was 0.412 nm, and the mean graphene crystallite size was 1.21 nm. The peaks at 38, 44.2, 64.3 and 77.3° can be indexed to (111), (200), (220) and (311) reflections of pure silver. The intensity of silver reflections is high, demonstrating that a large amount of silver nanoparticles were attached to the graphene sheets. The inset of Figure 1a presents the XRD pattern of graphene oxide, having the following structural characteristics: the number of layers is 8, the interlayer distance is 0.81 nm and the crystallite size is 6.76 nm.

To evidence the reduction of oxygen containing species during synthesis, the FTIR spectra of GO and Gr-Ag (5 wt% Ag) samples were measured (Figure 1b). In the spectrum of GO a large number of hydroxyl, carboxyl and epoxy groups are present, as pointed out by the characteristic peaks of the C=O stretching vibration at 1716 cm^−1^, the O-H deformation at 1384 cm^−1^, the C-O (epoxy) stretching vibration at 1228 cm^−1^, the C-O (alcoxy) stretching vibration at 1052 cm^−1^. The sp^2^ graphitic domains give signature at 1625 cm^−1^ in both FTIR spectra. After reduction and decoration of graphene oxide with silver nanoparticles, a decrease in the intensity of the oxygen-containing functional groups was noticed (especially C=O and O-H groups) along with the rise of a new peak at 1544 cm^−1^, due to C-C skeletal vibration in reduced graphene oxide sheets.

Using Raman spectroscopy, we evaluated the degree of structural disorder within graphene oxide and graphene decorated with silver nanoparticles (Figure 1c). In both samples, three important bands, characteristic to graphene-based materials, are evidenced: the defect (D) band at ~1345 cm^−1^, the graphite (G) band located at ~1590 cm^−1^ and the 2D band present at ~2700 cm^−1^. It is notable that after the reduction and decoration of graphene oxide with silver nanoparticles, the D and G peaks shifted. This can be attributed to the n-type doping of graphene induced by the decoration with AgNPs [31]. The I_D_/I_G_ intensity ratio gives an indication of the defect-free domains within the two samples: 0.99 nm for GO and 1.057 nm for Gr-Ag (5 wt% Ag) hybrid. The higher I_D_/I_G_ intensity ratio in Gr-Ag (5 wt% Ag) sample in comparison with that of GO may be attributed to more defects generated by silver decoration, resulting in highly disordered graphene nanosheets.

### 3.3. Microbiological Investigation of HEPA Filter Modified with Gr-Ag (5 wt% Ag) Hybrid Material

**Figure 2:**
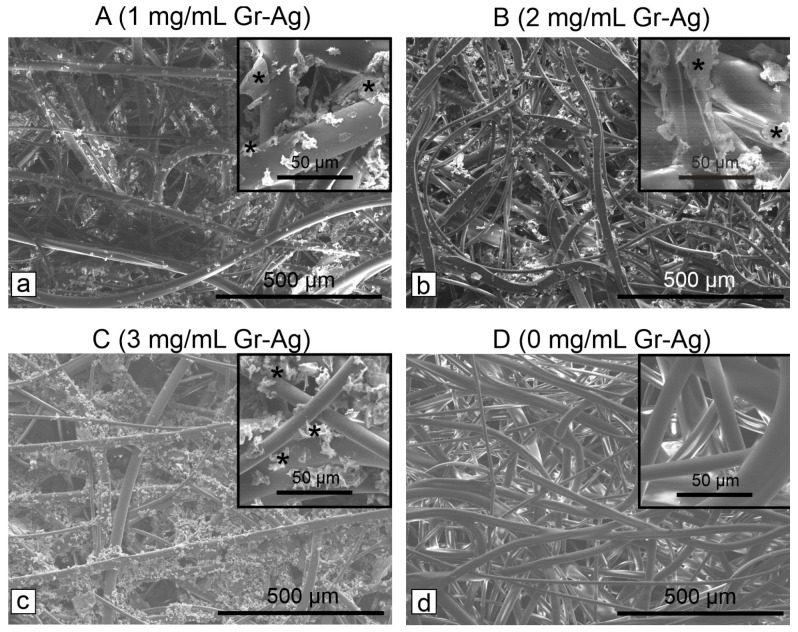
SEM micrographs showing the presence of the Gr-Ag (5 wt% Ag) hybrid (marked with *) attached to the HEPA filters (**a**–**c**), compared to the control filter (**d**) treated only with ethanol.

The filters modified with various concentrations of Gr-Ag (5 wt% Ag) were denoted as following: A (1 mg/mL), B (2 mg/mL) and C (3 mg/mL), while the control material was annotated as D (Figure 2). The results showed that the HEPA filters treated with 1 mg/mL Gr-Ag (5 wt% Ag) had no effect against *E. coli* strain, as well as the control material, compared to *S. aureus* strain, which showed sensitivity to all three treated materials (Table 1).

**Figure 3:**
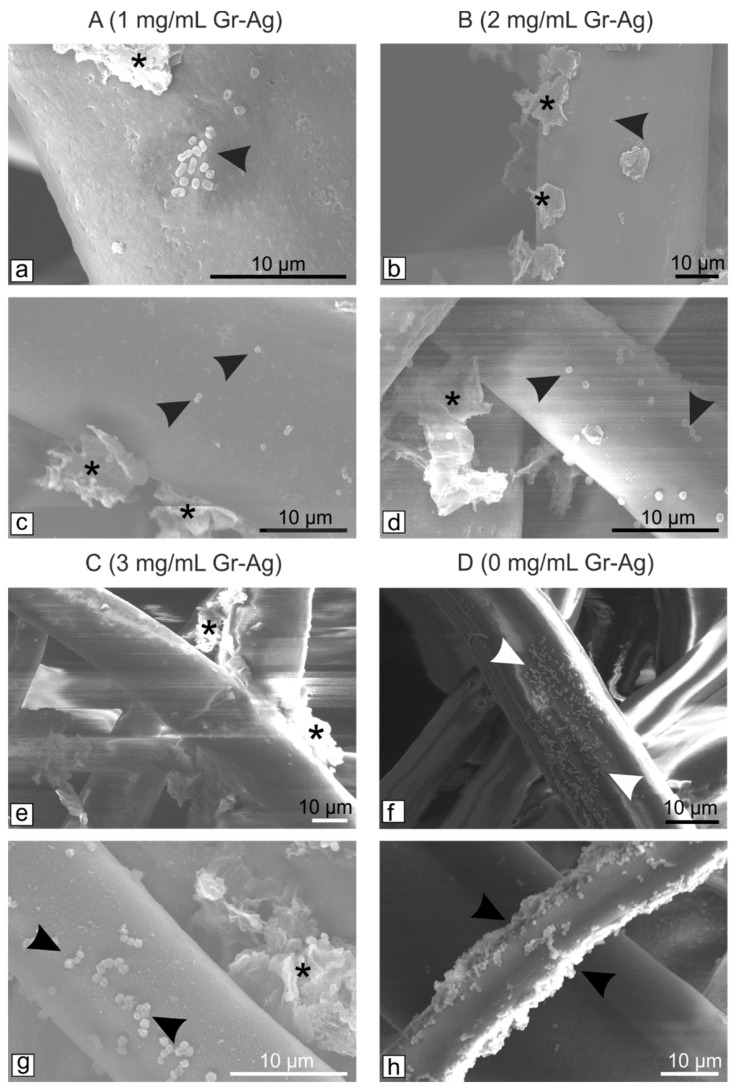
SEM micrographs of the HEPA filters treated with different concentrations of Gr-Ag (5 wt% Ag) hybrid (marked with *) and incubated with bacteria (marked with arrowheads) for 24 h at 37 °C: *E. coli* (**a**,**b**,**e**,**f**) and *S. aureus* (**c**,**d**,**g**,**h**).

**Figure 4:**
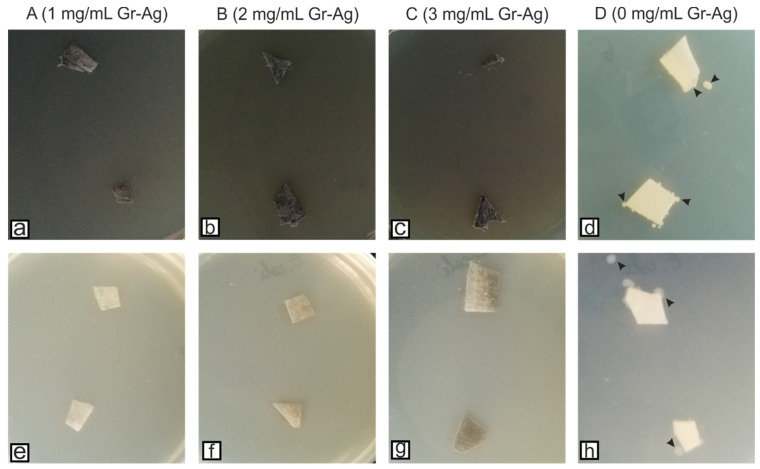
HEPA filters treated with different concentrations of Gr-Ag (5 wt% Ag) and the control filter incubated with *S. aureus* (**a**–**d**) and *E. coli* (**e**–**h**) for 24 h at 37 °C, and inoculated on fresh Mueller-Hinton agar media; black arrowheads indicate the presence of bacteria on the culture media (only the control filters had viable bacteria—D (0 mg/mL Gr-Ag).

According to the EUCAST protocols, at 0 colonies detected the effect is considered bactericidal, between 1 and 10^8^, bacteriostatic, and >10^8^, no effect. Since 0 colonies were observed for sample B, the 2 mg/mL concentration is considered the lowest concentration at which both bacterial strains are inhibited. However, the SEM images indicated that the bacteria remain attached to the filters (Figure 3), but most probably their viability is inhibited by the Gr-Ag (5 wt% Ag) hybrid (Figure 4), since no colonies were formed when the filters were placed on freshly prepared media.

**Figure 5:**
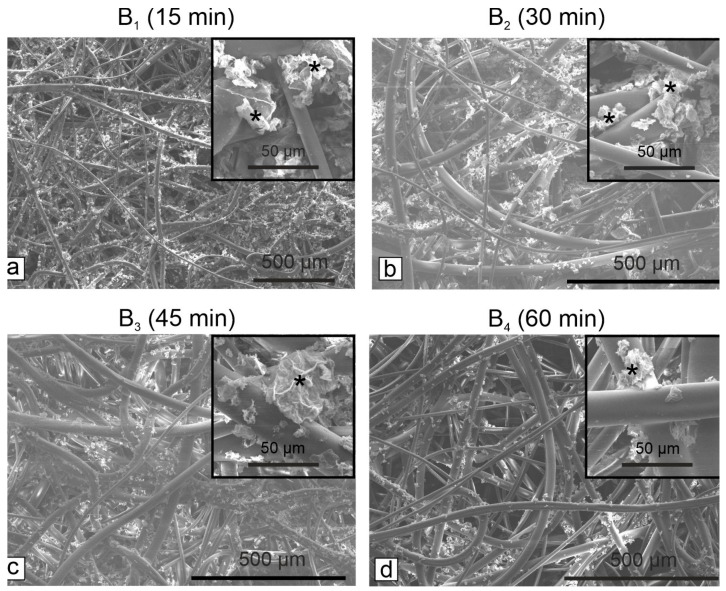
SEM micrographs of the HEPA filters treated with 2 mg/mL Gr-Ag (5 wt% Ag) hybrid (marked with *) for different time periods: (**a**)—15 min (B_1_), (**b**)—30 min (B_2_), (**c**)—45 min (B_3_), (**d**)—60 min (B_4_).

Based on the above measurements, we selected the optimum Gr-Ag (5 wt% Ag) concentration (2 mg/mL) and varied the immersion time: 15 (B_1_), 30 (B_2_), 45 (B_3_) and 60 (B_4_) min. The Gr-Ag (5 wt% Ag) loading on the HEPA filters was time-dependent, as indicated in Figure 5.

The CFU measurements showed that all tested materials had bactericidal effects against both Gram-negative and Gram-positive bacteria models (Table 2).

**Figure 6:**
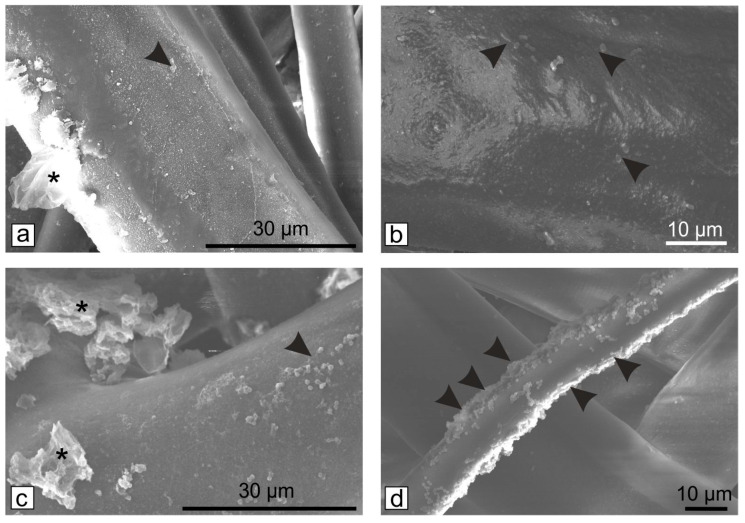
SEM micrographs of HEPA filters modified with 2 mg/mL Gr-Ag (5 wt% Ag) hybrid (asterisk) for 60 min and incubated with *E. coli* (**a**,**b**) and *S. aureus* (**c**,**d**) for 24 h at 37 °C; black arrowheads indicate the presence of bacteria on the fibers; bare HEPA filters (**b**–**d**).

Again, the filters were examined using SEM to determine if the bacteria remained attached to the fibers. Considering that all samples had bactericidal effect, B_4_ was the only filter examined along with the untreated control (Figure 6).

**Figure 7:**
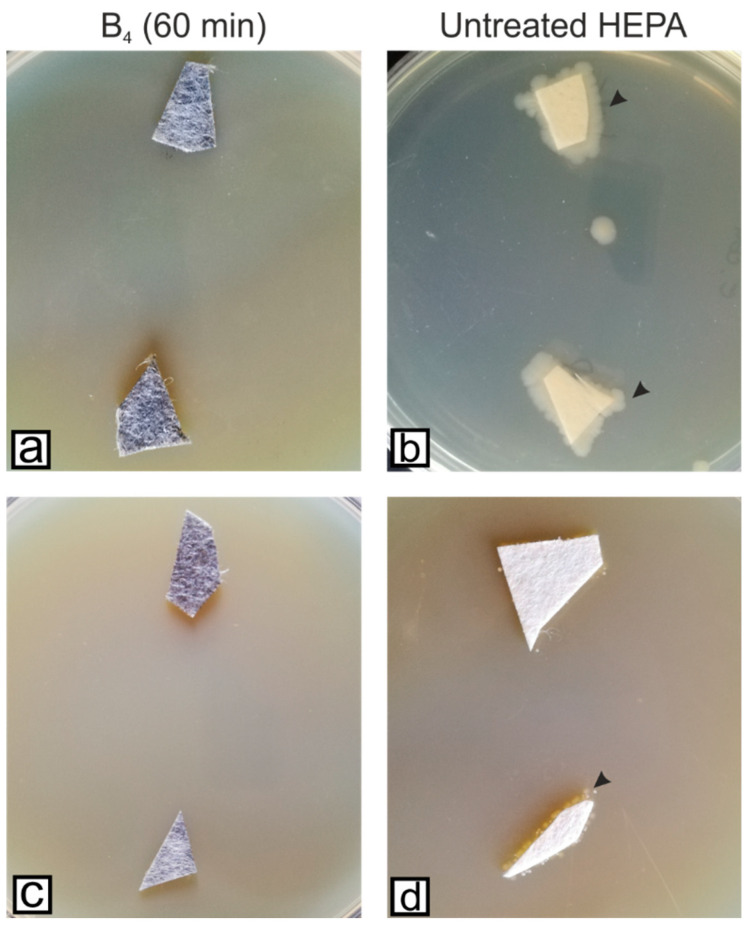
HEPA filters treated with 2 mg/mL Gr-Ag (5 wt% Ag) hybrid for 60 min and the control filter incubated with *E. coli* (**a**,**b**) and *S. aureus* (**c**,**d**) for 24 h at 37 °C, and inoculated on fresh Mueller-Hinton agar media; black arrowheads indicate the presence of bacteria on the culture media (only the untreated HEPA developed bacterial colonies).

Compared to the first tested filters (Figure 4), longer exposure to the Gr-Ag (5 wt% Ag) hybrid inhibited the attachment of the bacteria. Their viability was once again tested, and samples of the materials were placed on fresh MH agar media for 24 h, at 37 °C (Figure 7).

## 4. Discussion

The Gr-Ag (5 wt% Ag) hybrid material was morphologically and structurally characterized by advanced techniques (TEM, XRD, FTIR and Raman), which revealed the presence of silver nanoparticles attached to the thin graphene flakes. Further analyses were conducted to assess the antibacterial properties of the hybrid material. The effects were greater against Gram-negative bacteria, compared to the Gram-positive strain (*p* < 0.01). The average inhibition zone observed against *E. coli* was >19 mm, compared to *S. aureus* where the formed halo had an average of >8 mm. Similar results were reported by Joshi et al. (2020), when reduced graphene oxide conjugated with peptides were used against *E. coli* [12]. Truong et al. (2020) tested a similar compound and observed a better antibacterial effect against *E. coli*, compared to *S. aureus* [32]. However, other studies reported a greater inhibition of *S. aureus* than *E. coli*, when graphene oxide coupled with silver nanoparticles was tested [33,34].

Next, the HEPA filters were analyzed through SEM to determine the optimum hybrid material concentration and action time, necessary to inhibit the growth and development of bacterial strains. The longer the Gr-Ag (5 wt% Ag) hybrid was kept on the HEPA filters, the higher the loading was observed between the fibers and on the surface (Figure 5). Previous studies showed that bacteria could stay attached to membranes [35] or in this case to the filters, but the use of Gr-Ag (5 wt% Ag) hybrid reduced the viability of the strains, by different means of interactions between the graphene, silver, and bacteria [36].

Based on the previous findings, we can explain how the obtained hybrid affected the tested bacteria. The sharp-edge configuration of graphene can induce perturbations of the bacterial membranes, causing ruptures [37,38]. The different surface charges of bacterial membranes compared to Gr-Ag hybrid material enhances bacterial attachment. Once this happens, the sharp edge of graphene could modify the external protection of bacteria, inducing further intracellular damage [39]. For the mode of action of AgNPs, several explanations are currently formulated: cellular adhesion and penetration, disruption of the respiratory chain and cell signaling, and interfering with the biomolecular functions [40]. Gram-positive bacteria (*S. aureus*) have multiple peptidoglycan layers and a cell wall that acts as a barrier. Gram-negative bacteria (*E. coli*) enhance their protection through extra layers of lipopolysaccharides outer membranes, which make them generally more difficult to destroy [41]. Further on, if internalized, small AgNPs or Ag^+^ ions affect the bacterial respiratory system and ATP synthesis is inhibited by the formation of complexes between Ag^+^ ions and electron donor groups (various proteins), along with induction of reactive oxygens species, causing oxidative stress and eventually bacterial cell death [40].

It is important to emphasize that after modification of HEPA filter with Gr-Ag (5% Ag) material, its mechanical properties (strength and flexibility) were well preserved. The optical images (Figure S4) clearly evidenced that the filter was not damage after modification and was successfully tested in an experimental device, for air filtration. Hence, the modification of an existing technology such as HEPA filter, with graphene-silver hybrid, was shown to have an important role regarding the antibacterial properties. Therefore, this existing technology could be improved as the hybrid material obtained herein has a high potential for industrial usage.

## 5. Conclusions

The modification of HEPA filter with Gr-Ag (5 wt% Ag) hybrid material was conducted herein. The morphological characterization of the material showed the successful formation of the hybrid, results confirmed by XRD, FTIR and Raman as well. After the antibacterial characterization, different concentrations of the hybrid material were incubated with HEPA filters, and their bactericidal and bacteriostatic properties were measured. The optimum Gr-Ag concentration was chosen based on the observed results, and once again the HEPA filters were incubated with the hybrid material for different time periods. The results showed that both *E. coli* and *S. aureus* remain attached to the HEPA filters, but compared to the untreated controls, their density differs, and the viability is affected by the presence of Gr-Ag (5 wt% Ag) hybrid.

## Figures and Tables

**Table 1:** The colony forming units (CFU/mL) method indicating the bactericidal/bacteriostatic effect of the HEPA filter treated with different concentrations of Gr-Ag (5 wt% Ag) hybrid.

Materials	CFU/mL	
	*E. coli*	*S. aureus*
A	>10^10^	0
B	0	0
C	0	0
D	>10^10^	6 × 10^10^
Control	>10^10^	3 × 10^10^

A = 1 mg/mL Gr-Ag (5 wt% Ag); B = 2 mg/mL Gr-Ag (5 wt% Ag); C = 3 mg/mL Gr-Ag (5 wt% Ag); D = control material treated with pure ethanol; Control = untreated bacterial suspension.

**Table 2:** The colony forming units (CFU/mL) method indicating the bactericidal/bacteriostatic effect of the HEPA filters treated with 2 mg/mL of Gr-Ag (5 wt% Ag) hybrid for different time periods.

Materials	CFU/mL	
	*E. coli*	*S. aureus*
B_1_	0	0
B_2_	0	0
B_3_	0	0
B_4_	0	0
Untreated HEPA	>10^10^	>10^10^

B_1_ = 15 min treatment, B_2_ = 30 min treatment, B_3_ = 45 min treatment, B_4_ = 60 min treatment, Untreated HEPA = HEPA material immersed in pure ethanol for 1 h.

## Data Availability

Data will be provided upon reasonable request to the corresponding author.

## Supplementary Materials

The following supporting information can be downloaded at: https://www.mdpi.com/article/10.3390/microorganisms11030745/s1, Figure S1: Representative TEM micrographs of reduced graphene oxide with no nanoparticles attached on its surface (**a**,**b**); Figure S2: Representative TEM micrographs of Gr-Ag hybrids with various quantities of Ag and their corresponding histograms depicting the size distribution of nanoparticles: Gr-Ag (3 wt% Ag; **a**,**b**); Gr-Ag (5 wt% Ag; **c**,**d**); and Gr-Ag (20 wt% Ag; **e**,**f**); Figure S3: The antibacterial assessment against *S. aureus* and *E. coli* of rGO, AgNPs and Gr-Ag hybrids with different quantities of Ag; * *p* < 0.05, ** *p* < 0.01, *** *p* < 0.001 according to Students’ *t* test. Figure S4: Optical images of HEPA filters before and after modification with Gr-Ag (5% Ag) material.
