# Supplementary material for: Antibacterial Enhancement of High-Efficiency Particulate Air Filters Modified with Graphene-Silver Hybrid Material
Source: Microorganisms. 2023 Mar 14;11(3):745. doi: 10.3390/microorganisms11030745 (PMC10059912; doi:10.3390/microorganisms11030745)
Supplement: Supplementary file 1 [file microorganisms-11-00745-s001.zip › microorganisms-2239070-supplementary.pdf]

Supporting Information Material

Antibacterial Enhancement of High-Efficiency Particulate Air Filters Modified with Graphene-Silver Hybrid Material

Alexandra Ciorîță [1,2,*], Maria Suciu [1,2], Maria Coroş [2], Codruța Varodi [2], Florina Pogăcean [2], Lidia Măgeruşan [2], Valentin Mirel [2], Raluca-Ioana Ștefan-van Staden [3] and Stela Pruneanu [2,*]

[1] Faculty of Biology and Geology, Babeș-Bolyai University, 44 Republicii Street, 400015 Cluj-Napoca, Romania
[2] National Institute for Research and Development of Isotopic and Molecular Technologies, 67-103, Donat Street, 400293 Cluj-Napoca, Romania
[3] Laboratory of Electrochemistry and PATLAB, National Institute of Research for Electrochemistry and Condensed Matter, 202 Splaiul Independentei Street, 060021 Bucharest, Romania
* Correspondence: alexandra.ciorita@itim-cj.ro (A.C.); stela.pruneanu@itim-cj.ro (S.P.)

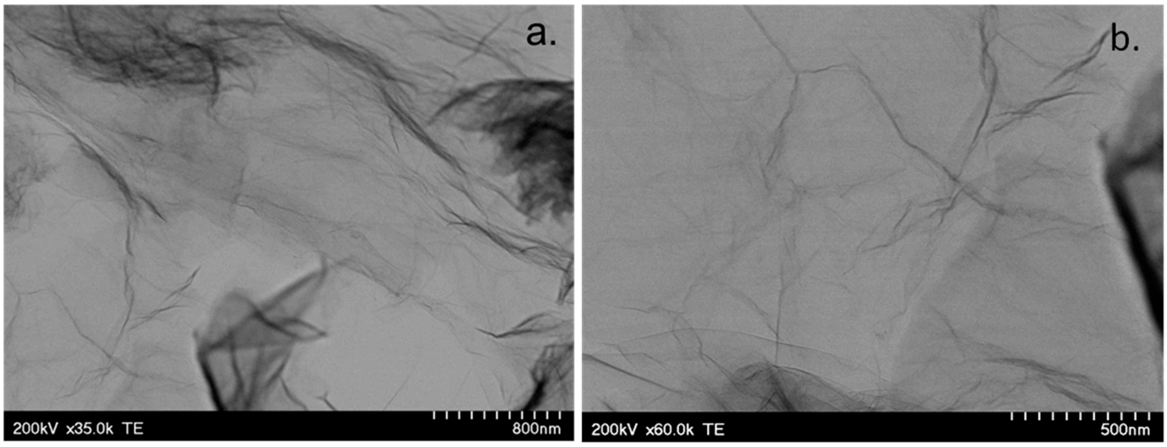

Figure S1. Representative TEM micrographs of reduced graphene oxide with no nanoparticles attached on its surface (a, b).

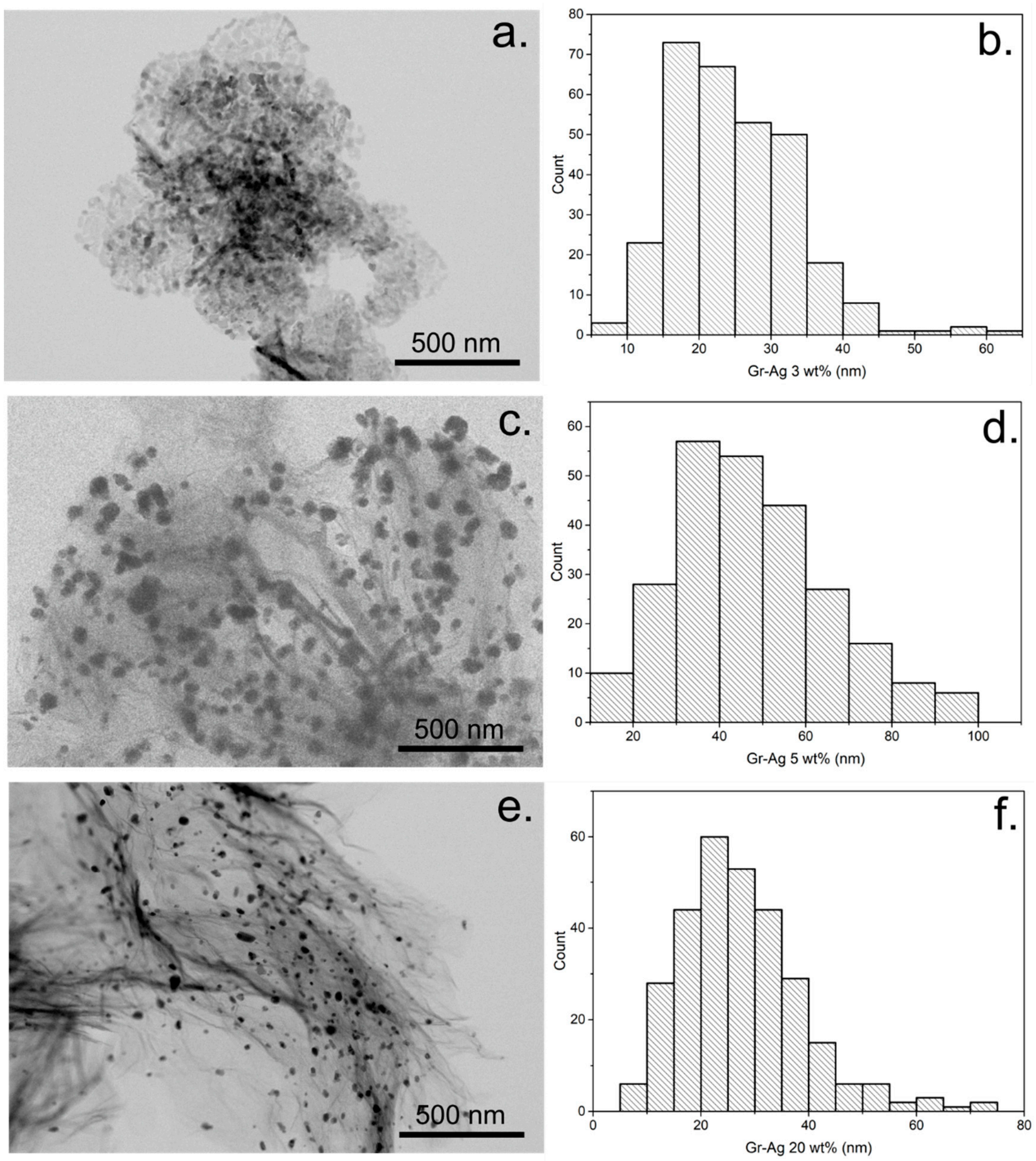

Figure S2. Representative TEM micrographs of Gr-Ag hybrids with various quantities of Ag and their corresponding histograms depicting the size distribution of nanoparticles: Gr-Ag (3 wt% Ag; a-b); Gr-Ag (5 wt% Ag; c-d); and Gr-Ag (20 wt% Ag; e-f).

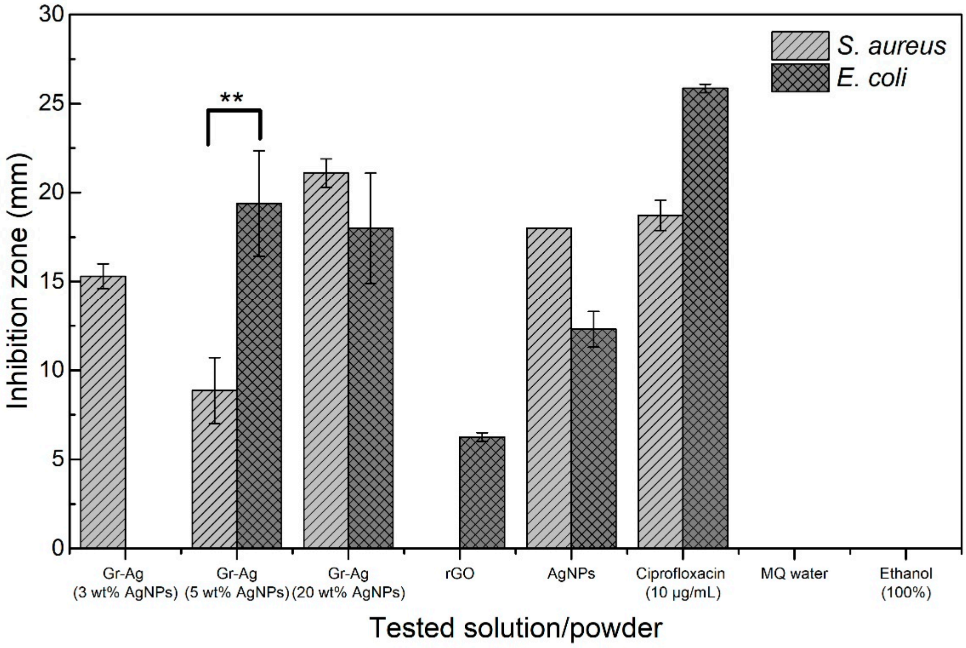

Figure S3. The antibacterial assessment against *S. aureus* and *E. coli* of rGO, AgNPs and Gr-Ag hybrids with different quantities of Ag; * $p<0.05$, ** $p<0.01$, *** $p<0.001$ according to Students' *t* test.

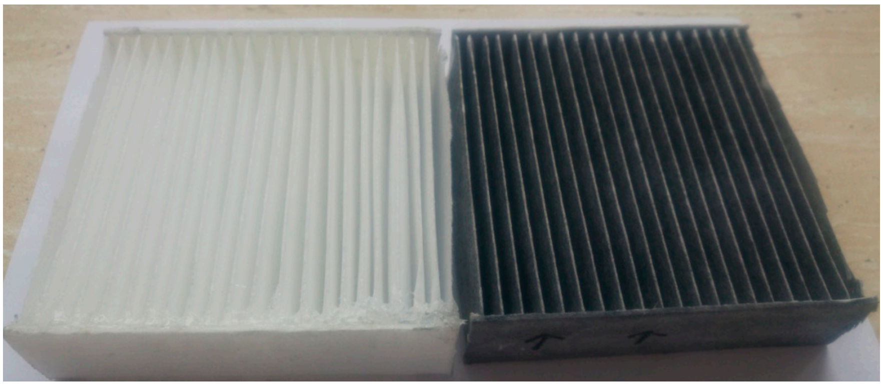

Figure S4. Optical images of HEPA filters before and after modification with Gr-Ag (5% Ag) material.
